# Potential clinical implications of molecular mimicry‐induced autoimmunity

**DOI:** 10.1002/iid3.1178

**Published:** 2024-02-01

**Authors:** Bandar A Suliman

**Affiliations:** ^1^ Department of Clinical Laboratory Sciences, College of Applied Medical Sciences Taibah University Madinah Saudi Arabia

**Keywords:** autoimmunity, epitopes

## Abstract

**Background:**

Molecular mimicry is hypothesized to be a mechanism by which autoimmune diseases are triggered. It refers to sequence or structural homology between foreign antigens and self‐antigens, which can activate cross‐reactive lymphocytes that attack host tissues. Elucidating the role of molecular mimicry in human autoimmunity could have important clinical implications.

**Objective:**

To review evidence for the role of molecular mimicry in major autoimmune diseases and discuss potential clinical implications.

**Methods:**

Comprehensive literature review of clinical trials, observational studies, animal models, and immunology studies on molecular mimicry in multiple sclerosis, type 1 diabetes, rheumatoid arthritis, lupus, Guillain‐Barre syndrome, autoimmune myocarditis, and primary biliary cirrhosis published from 2000–2023.

**Results:**

Substantial indirect evidence supports molecular mimicry as a contributor to loss of self‐tolerance in several autoimmune conditions. Proposed microbial triggers include Epstein‐Barr virus, coxsackievirus, Campylobacter jejuni, and bacterial commensals. Key mechanisms involve cross‐reactive T cells and autoantibodies induced by epitope homology between microbial and self‐antigens. Perpetuation of autoimmunity involves epitope spreading, inflammatory mediators, and genetic factors.

**Conclusions:**

Molecular mimicry plausibly explains initial stages of autoimmune pathogenesis induced by infection or microbiota disturbances. Understanding mimicry antigens and pathways could enable improved prediction, monitoring, and antigen‐specific immunotherapy for autoimmune disorders. However, definitive proof of causation in humans remains limited. Further research should focus on establishing clinical evidence and utility.

## 1 INTRODUCTION

The immune system has evolved to protect us against infectious pathogens and other foreign antigens using innate and adaptive mechanisms. However, in some individuals, dysregulation of the immune response can occur, leading to intolerance towards self‐antigens and subsequent tissue damage known as autoimmunity. A broad range of inflammatory and autoimmune disorders are driven by inappropriate reactions against the body's own components. Elucidating mechanisms by which tolerance breaks down and pathogenic autoimmunity arises remains key for developing diagnostic and therapeutic approaches. Molecular mimicry has emerged as a prominent mechanistic hypothesis to explain the initial triggering of misdirected immune responses against self‐antigens in many autoimmune diseases.

Inflammation forms a core part of the normal immune response to harmful stimuli like invading microbes. Detection of pathogen‐associated molecular patterns on bacteria, viruses, or parasites by innate pattern recognition receptors triggers secretion of proinflammatory cytokines and chemokines that recruit neutrophils, dendritic cells, and other immune cells to the site of infection or injury. Adaptive immunity centered on antigen‐specific T and B lymphocytes provides more targeted, albeit delayed, responses to pathogens based on recognition of unique antigenic epitopes. Elimination of the antigen source along with inhibitory receptors, regulatory cells, and anti‐inflammatory mediators helps resolve productive immunity and prevent excessive inflammation.

However, in some circumstances this controlled response becomes dysregulated, resulting in chronic inflammation or misdirected reactivity against self‐components, known as autoimmunity. Genetic polymorphisms, environmental exposures, hormonal influences, and infections can all contribute to breakdown of immune tolerance. Our current understanding proposes that interactions between susceptible genes and environmental triggers initiate the earliest stages of autoimmunity, which is subsequently propagated by additional secondary factors. Identifying these initial triggers and inciting antigens is key for developing preventive and early treatment strategies.

In this context, molecular mimicry has emerged as a leading mechanistic hypothesis by which microbial pathogens or other environmental stimuli can provoke loss of tolerance and activation of self‐reactive lymphocytes. Molecular mimicry refers to sequence or structural homology between exogenous antigens and self‐antigens. Foreign antigens that mimic or closely resemble epitopes on native human proteins can stimulate cross‐reactive lymphocytes that inadvertently attack host tissue expressing the mimicked self‐antigen. Such T or B cell epitope mimicry provides a plausible basis by which infection, microbiota shifts, or other immune stimuli could precipitate autoimmune pathology in genetically prone individuals via de novo activation of quiescent autoreactive clones.

Substantial evidence from human correlation studies, animal models, and in vitro experiments supports roles for molecular mimicry in triggering diverse autoimmune conditions like multiple sclerosis (MS), type 1 diabetes, rheumatoid arthritis, myocarditis, and others. However, conclusively proving causality in humans remains challenging. This literature review synthesizes current evidence regarding proposed molecular mimicry mechanisms across a spectrum of prominent autoimmune diseases. Pathophysiologic discussions for each condition highlight key findings related to potential environmental mimics and corresponding self‐antigen targets that provide the foundation for initial loss of tolerance. Examples of emerging research strategies to leverage knowledge of disease‐specific mimicry antigens and pathways for predictive, preventive and therapeutic purposes are also examined. Overall, elucidating molecular mimicry represents a promising avenue for better understanding early autoimmune triggers and harnessing this knowledge for clinical translation.

## 2 METHODOLOGY

A comprehensive literature search was conducted to examine the existing evidence for the role of molecular mimicry in various autoimmune diseases, evaluate proposed mechanisms, and identify relevant ongoing research from January 2000 to February 2023. The search focused on retrieving primary literature and review articles published in English language peer‐reviewed journals.

Multiple electronic databases were searched including PubMed, Embase, Scopus, Web of Science, and Google Scholar. The main search concepts used were the terms “molecular mimicry,” specific autoimmune disease names (e.g. “multiple sclerosis”), and terms indicating pathogenesis/mechanisms (e.g. “pathogenesis” and “etiology”). For each disease, searches combined terms for molecular mimicry and the disease name, along with filters for the date range. Autoimmune diseases examined included MS, type 1 diabetes, rheumatoid arthritis, systemic lupus erythematosus (SLE), Guillain‐Barre syndrome (GBS), autoimmune myocarditis, and primary biliary cirrhosis (PBC).

Retrieved abstracts were screened for relevance to molecular mimicry mechanisms and pathogenesis. Full‐text review was performed for pertinent original research providing direct or indirect evidence on the role of molecular mimicry and potential microbial triggers. Priority was given to human clinical studies as well as mechanistic studies in animal models with translational relevance. High‐quality reviews offered additional context and analysis. Relevant bibliographies were hand‐searched for additional studies. Experiments purely in vitro were given lower weight compared to in vivo data.

Searches focused on retrieving controlled trials, cohort studies, case‐control studies, and systematic reviews which provided the highest levels of evidence regarding disease mechanisms and molecular mimicry. For emerging research, preclinical studies and early phase human studies were also considered. Collectively, more than 130 full‐text articles across the categories of clinical trials, observational studies, review articles, animal models, and basic immunology studies provided the foundation for the literature review.

Data extracted from reviewed studies included proposed mechanisms of molecular mimicry, microbial antigens exhibiting homology with self‐proteins, targeted autoantigens, presence of cross‐reactive antibodies/T cells, animal model immunizations demonstrating induced autoimmunity via mimicry, and epidemiological or bioinformatic associations between infections and autoantigens. Identified mechanisms were analyzed to develop an overview of molecular mimicry hypotheses, experimental support, limitations, unknowns, and areas of promise for future research and clinical applications.

Based on the compiled evidence, detailed pathogenic discussions were constructed for each autoimmune disease highlighting proposed molecular mimicry mechanisms and key supportive findings. Discussions aimed to focus on human studies and translational models regarding potential mimic antigens, but also summarized in vitro data and bioinformatic associations when direct in vivo evidence was limited. The involvement of both humoral and cellular immunity in autoimmune processes mediated or propagated by mimicry was synthesized.

Follow‐up searches were conducted to identify active research avenues and early‐stage clinical trials related to leveraging knowledge of molecular mimicry triggers for antigen‐specific immunomodulatory therapies. This provided examples of approaches under study to translate mechanistic concepts of mimicry into clinical applications for predicting, preventing, or treating specific autoimmune conditions. Literature searches were supplemented by manually reviewing citations to obtain additional relevant sources.

In summary, a systematic search of multiple literature databases over a 20+ year period was conducted to compile quality evidence from human, animal, and in vitro studies on molecular mimicry mechanisms across a spectrum of prominent autoimmune diseases. Both primary literature and expert syntheses were analyzed to generate comprehensive pathogenic discussions and provide illustrative examples of ongoing efforts to therapeutically target disease‐specific molecular mimicry triggers and pathways. This rigorous approach aimed to support an evidence‐based examination of current knowledge and future directions related to molecular mimicry and autoimmunity.

## 3 AN EVOLUTIONARY MALFUNCTION OF THE IMMUNE SYSTEM

Molecular mimicry can lead to immune repose against self‐antigens due the similarity with a non‐self‐antigen exists in a foreign body. The immune system is supposed to protect the body by distinguishing “self” from “foreign” and targeting potentially harmful pathogens. Molecular mimicry undermines this distinction, leading the immune system to mistakenly attack the body's own tissues. From an evolutionary standpoint, however, the emergence of molecular mimicry may not represent a “failure,” but rather an unavoidable trade‐off of having an adaptive immune system.

On one hand, molecular mimicry reflects the challenges of the immune system to distinguish between self and non‐self‐due to its need for a broad range of antigen recognition. The immune system relies on generating tremendous diversity among antibodies and T‐cell receptors through genetic recombination. This allows recognition of a vast array of antigenic determinants.^1^ However, it also inevitably leads to some degree of cross‐reactivity between foreign and self‐antigens. Molecular mimicry exemplifies this imperfect specificity. In this light, it could be considered an unintended consequence or shortcoming of the adaptive immune system's flexibility.

On the other hand, molecular mimicry may confer some evolutionary benefits that offset its potential costs. Cross‐reactivity expands the immune system's antigen detection capabilities, enabling it to rapidly respond to pathogens it has never encountered before but that share sequences with known antigens.^2^ This built‐in “degeneracy” likely enhances immunological preparedness against unpredictable microbial attacks. Furthermore, autoimmunity mediated by molecular mimicry may help amplify immune responses to specific pathogens. The resulting inflammation, while damaging to host tissues, could aid in pathogen clearance.

Additionally, molecular mimicry‐induced autoimmunity likely only represents the initial trigger that precipitates loss of tolerance. For clinically apparent autoimmune disease to manifest, this early breach in self‐tolerance requires additional facilitating factors. Sustained autoimmunity probably results from complex interactions between the molecular mimicry trigger, genetic susceptibilities like HLA alleles, and influences from the environmental milieu.^3^ In other words, molecular mimicry on its own is unlikely to be solely responsible for overt autoimmune disease in most individuals. Therefore, from a population genetics perspective, the risks of molecular mimicry may be outweighed by its potential survival advantages.^4^

While molecular mimicry can lead to harmful autoimmunity, it may be an inevitable evolutionary consequence of generating a diverse antigen receptor repertoire capable of recognizing innumerable pathogens.^5^ The presence of regulatory T cells and mechanisms of immune tolerance also help minimize the risks of molecular mimicry‐induced autoimmunity. From a balanced perspective, molecular mimicry does not necessarily represent an actual malfunction or failure of the immune system but rather a delicate trade‐off between robust antimicrobial immunity and avoidance of self‐reactivity. This trade‐off appears to have been well‐tolerated over long evolutionary timescales. An intensive understanding of molecular mimicry may inform techniques to selectively manipulate cross‐reactivity to medicine's advantage.

## 4 AN EDGE FOR PATHOGENS OVER THE IMMUNE SYSTEM

It may seem evident that molecular mimicry gives pathogens an upper hand in their evolutionary arms race with humans. By disguising parts of themselves as “self,” pathogens trick the immune system into tolerance, thereby promoting microbial survival and replication. However, the advantages of molecular mimicry to pathogens are more nuanced upon closer examination.

Molecular mimicry does appear to enable certain pathogens to partly evade or subvert immune detection. Mimicry of host proteins allows these organisms to hide critical pathogenic components that would normally elicit strong immune attack. For example, some strains of Streptococcus bacteria mimic collagen, enabling them to inhibit phagocytic clearance.^6^ Human cytomegalovirus camouflages its glycoproteins by mimicking MHC molecules, obstructing antiviral responses.^7^ Such mimicry mechanisms are thought to have evolved through selective pressures to escape immune elimination.

However, molecular mimicry also carries significant risks and downsides from the pathogen perspective. Inducing autoimmune reactions may elicit inflammation that helps mobilize multifaceted antimicrobial responses. Viruses that trigger autoimmunity through mimicry can essentially co‐opt the immune system into promoting their own clearance.^8^ Additionally, many pathogens rely on infecting host cells to survive and proliferate. Autoimmune destruction of the host tends to undermine pathogen fitness and transmission. So rampant molecular mimicry would seem counterproductive for pathogens dependent on host viability.

Furthermore, molecular mimicry does not guarantee immune tolerance and often still provokes an immune response.^9^ Highly inflammatory reactions triggered by mimic antigens may override tolerance and allow pathogens displaying molecular mimicry to be eliminated.^10^ The optimal level of mimicry likely balances avoidance of immune attack with continued ability to productively infect host cells.

Lastly, molecular mimicry is just one of numerous pathogen immune evasion and virulence strategies. Pathogens do not rely solely on molecular mimicry and have evolved a variety of other mechanisms to enhance their survival and propagation. Molecular mimicry should therefore be considered just one component of the complex host‐pathogen interaction.

While providing some advantage to certain pathogens in specific contexts, molecular mimicry also has limitations and drawbacks from an evolutionary standpoint. Molecular mimicry alone is rarely enough to enable unchecked microbial growth and completely thwart immune defenses. Its relative benefits are pathogen‐specific and contingent on many factors.^4^, ^11^ Overall, categorizing molecular mimicry as conferring a decisive advantage to pathogens across the board appears to be an oversimplification. A balanced view recognizes molecular mimicry as an intriguing pathogen strategy, but one that comes with both costs and benefits in the unending arms race between microbes and immune systems.

## 5 MS

MS is an autoimmune disorder characterized by inflammatory damage to myelin, oligodendrocytes, and axons in the central nervous system (CNS). Pathophysiology involves activating self‐reactive T and B cells that target and destroy myelin, leading to sclerotic plaque formation and neurological impairments.^12^ While the exact triggers of MS remain unknown, molecular mimicry between components of various pathogens and CNS antigens is a prominent mechanistic hypothesis for initiating autoreactive immune responses.

Molecular mimicry posits that sequence or structural homologies between exogenous microbial antigens and self‐antigens can stimulate cross‐reactive lymphocytes that, in turn, attack CNS targets like myelin basic proteins, proteolipid proteins, and myelin oligodendrocyte glycoproteins. Both viral and bacterial antigens have been proposed as potential mimicry‐inducing agents.^13^ For example, Epstein‐Barr virus (EBV) proteins share short amino acid stretches with myelin proteins that may activate cross‐reactive T cells.^14^ Other viruses like human herpesvirus‐6^15^ and influenza virus^16^ also contain epitopes mimicking myelin components. On the bacterial side, mimicry with CNS antigens has been reported for Hemophilus influenzae,^17^ Mycobacterium tuberculosis,^18^ and Acinetobacter baumannii proteins.^19^

According to the molecular mimicry model, exposure to such pathogens through infection or microbiota stimulation induces an immune response containing B cells and T cells that cross‐react with myelin determinants in genetically susceptible individuals. The aberrant autoreactive lymphocytes then migrate to the CNS, initiating autoimmune demyelination and neurodegeneration.^20^ CD4+ and CD8+ T cells that react with myelin epitopes have been detected in MS patients, supporting this mechanistic pathway.^21^ B cells producing antibodies recognizing mimicry antigens and myelin components may contribute to myelin damage through opsonization.^22^

While molecular mimicry is a long‐standing hypothesis for the earliest stages of MS pathogenesis, CNS inflammation, and demyelination subsequently provoke responses that perpetuate and amplify the autoimmune response. These include epitope spreading, activation of microglia and macrophages, complement activation, and formation of ectopic B cell follicles in the CNS.^23^ Epitope spreading refers to the diversification of the autoimmune reaction to target additional myelin epitopes beyond the original cross‐reactive antigenic site. As myelin injury exposes more self‐antigens, this further expands and matures the autoreactive T cell and B cell repertoire.^24^

Molecular mimicry is believed to be the initial trigger that activates self‐reactive lymphocytes and sets the stage for CNS autoimmunity. However, sustained autoimmune‐mediated demyelination requires a breach of tolerance mechanisms and actions of other neuroinflammatory mediators like microglia, astrocytes, and innate immune cells, which perpetuate the destructive process. Both arms of adaptive immunity (T and B cells) coordinate to drive chronic inflammatory lesions and eventual axonal damage. The result is plaques of demyelination and scarring that cause progressive neurological disability.

Although molecular mimicry has long been hypothesized as a mechanism capable of instigating harmful immune reactions against myelin, conclusive evidence to validate this proposed role in initiating anti‐myelin autoimmunity is still to be elucidated. The specific microbes and sequence homologies responsible for triggering mimicry in most MS patients remain undetermined.^25^ However, indirect evidence from human studies and experimental animal models supports the viability of molecular mimicry as one contributing factor in MS pathophysiology. Further research to identify the critical mimicry antigens and understand their interactions with susceptibility genes may enable the development of antigen‐specific immunotherapies. Overall, molecular mimicry remains a compelling model to partly explain the aberrant autoimmune targeting of myelin antigens that drive inflammation, demyelination, and neurodegeneration in MS.

Until today, few trials have been testing whether immunization with EBV or human herpesvirus‐6‐derived peptides can induce tolerance and reduce disease activity in MS patients. The antigens used are suspected viral mimics of myelin proteins.^26^ Other trials used synthetic myelin peptides such as myelin basic protein, myelin oligodendrocyte glycoprotein, and proteolipid protein to tolerate myelin‐reactive T cells. Some approaches involved administering myelin peptides via intravenous, oral, or intranasal routes.^27^ Researchers are also studying DNA vaccines encoding myelin antigens to induce tolerance while avoiding general immunosuppression selectively.^28^ Antigen‐coupled cells, such as red blood cells bound to myelin peptides, are being tested to induce regulatory T cell responses against myelin‐reactive immune cells.^29^ Ongoing efforts include identifying immunodominant myelin T cell epitopes and testing modified peptide antigens that retain immunogenicity but reduce stimulatory capacity.^30^ B cell epitope‐specific therapies using antibodies against the pathogenic parts of myelin antigens are also under investigation.^31^ Combination approaches use myelin antigen immunization and molecules that stimulate regulatory T cells or myelin‐protective immune responses.^32^

## 6 TYPE‐1 DIABETES MELLITUS (T1DM)

T1DM is an autoimmune disease characterized by T cell‐mediated destruction of the insulin‐producing beta cells in the pancreatic islets of Langerhans. This leads to insulin deficiency and hyperglycemia. The pathophysiology of type 1 diabetes involves both cellular and humoral autoimmunity. Key target antigens expressed in pancreatic beta cells that are attacked in the autoreactive responses include insulin, glutamic acid decarboxylase (GAD), islet antigen‐2 (IA‐2), and zinc transporter 8 (ZnT8).^33^ Molecular mimicry between viral antigens and self‐antigens has been implicated as a possible mechanism for the initial triggering of autoreactive T lymphocytes that drive the autoimmune process.^34^

Several lines of evidence support molecular mimicry in T1DM pathogenesis. For example, coxsackievirus infection has been linked epidemiologically to the subsequent development of T1DM.^35^ At the molecular level, coxsackievirus P2‐C protein shares sequence homology with GAD65, a major autoantigen in T1DM.^36^ This epitope similarity may stimulate cross‐reactive T cells that recognize both viral and GAD65 peptides, initiating anti‐islet autoimmunity in genetically predisposed individuals. Homologies with GAD65 have also been found in rotavirus VP7 protein, further implicating molecular mimicry with pancreatic antigens.^37^

Besides viruses, mimicry between gut bacterial proteins and islet antigens has also been proposed to generate cross‐reactive T cells involved in beta cell destruction. Bacteroides thetaiotaomicron and Lactobacillus species express proteins that contain amino acid sequences shared with IA‐2 and ZnT8 diabetes autoantigens.^37^ This molecular homology may allow intestinal microbes to induce T‐cell responses that secondarily target the pancreas.^38^

According to the molecular mimicry hypothesis, exposure to such environmental triggers in conjunction with specific HLA Class II alleles leads to activation of cross‐reactive CD4+ T helper cells in the pancreatic lymph nodes. These T cells are then primed to attack islet cells presenting peptides from GAD65, IA‐2, insulin, and other beta‐cell proteins.^37^ As T cell‐mediated injury causes beta cell death, further islet antigens are released, stimulating epitope spreading. B cells are also activated to produce autoantibodies against insulin and GAD65. The result is a destructive immune infiltrate rich in autoreactive CD4+ and CD8+ T cells, inflammatory macrophages, dendritic cells, and B cells within the pancreatic islets.^39^

While molecular mimicry potentially explains the initial priming of pathogenic T cells, other mechanisms propagate the autoimmune response and drive clinical disease. These include bystander activation of T cells, defective immunoregulation, imbalance between regulatory and effector T cells, and local release of inflammatory mediators.^40^ In particular, defects in natural T regulatory cells that maintain self‐tolerance are thought to be essential to sustained autoimmunity in T1DM.^41^ The progressive loss of insulin secretion eventually leads to hyperglycemia once functional beta cell mass declines below a critical threshold.

In summary, molecular mimicry between viral or bacterial antigens and key islet autoantigens like GAD65 and IA‐2 may be responsible for triggering cross‐reactive T cells that initiate the autoinflammatory cascade targeting beta cells. However, the initial autoimmune trigger induced by molecular mimicry alone may not be sufficient for disease pathogenesis. Perpetuation of autoimmunity likely requires additional contributing factors like epitope spreading, local cytokine production, defective Treg function, and genetic risk variants.^42^ While direct proof of causative mimic antigens remains limited in humans, molecular mimicry represents a critical mechanistic hypothesis for the earliest inductive events leading to aberrant anti‐islet immunity in T1DM pathogenesis.^43^ Understanding these triggers may inform strategies for early prediction of T1DM risk and antigen‐specific immunomodulatory therapies.

Several clinical trials are testing insulin, proinsulin, and insulin peptide vaccines to induce immune tolerance to a key autoantigen involved in beta cell destruction.^44^ Several other trials are evaluating GAD65 vaccines using recombinant GAD65 protein or DNA encoding GAD65 to tolerate GAD65‐reactive T cells.^45^ Researchers are also testing diabetes vaccines using IA‐2 and ZnT8 peptides, alone or in combination, to dampen autoreactivity.^46^ Coxsackievirus vaccines are being explored to prevent mimicry‐induced activation of T cells targeting islet antigens like GAD65.^47^ Approaches using nanoparticles or immune‐modulating adjuvants to deliver islet antigens in a tolerogenic fashion are under investigation.^48^ Dendritic cell therapy using tolerogenic dendritic cells pulsed with islet antigens to delete or induce regulatory T cells is another strategy.^34^ Some other trials are combining islet antigen immunization with agents like vitamin D, rapamycin, or IL‐2 that enhance regulatory T cell activity.^49^

## 7 RHEUMATOID ARTHRITIS

Rheumatoid arthritis (RA) is an autoimmune disease characterized by chronic inflammation and destruction of synovial joints. Pathophysiology involves a breakdown in self‐tolerance leading to dysregulated immune responses against joint tissues. Both cellular and humoral arms of immunity contribute to pathogenic inflammation and cartilage/bone damage. Molecular mimicry between microbial antigens and self‐proteins has been hypothesized as a mechanism that could initiate an autoreactive response.

Several lines of evidence indicate EBV may be a source of mimicry antigens relevant to RA. EBV proteins share sequence homology with human collagen and keratin, which are targets of RA autoantibodies. Antibodies against the EBV nuclear antigen‐1 (EBNA‐1) cross‐react with peptides from proteoglycans critical for cartilage integrity.^50^ Sequence similarity between the EBV DNA polymerase and human filaggrin is also proposed to drive production of anti‐citrullinated protein antibodies (ACPA) highly specific to RA.^51^ Through such molecular mimicry, EBV could prime B cells and T cells that cross‐react with synovial joint antigens.^52^

According to the mimicry model, EBV infection in genetically susceptible individuals induces activation of autoreactive helper and killer T cells, as well as B cells producing rheumatoid factor, ACPA, and antibodies against joint collagen and proteoglycans.^53^ These autoreactive lymphocytes accumulate in the synovium and stimulate the release of inflammatory cytokines like TNF‐alpha, IL‐1beta, IL‐6, and IL‐17.^54^

Proinflammatory macrophage and fibroblast activation leads to synovial hyperplasia and osteoclast‐mediated bone erosion. Recruitment of immune cells from the circulation maintains chronic inflammation and drives cartilage degradation and joint destruction.

While molecular mimicry may provide the initial trigger, other factors propagate and amplify the autoimmune response. Sustained inflammation in the joints facilitates epitope spreading, wherein immune reactivity diversifies from the original cross‐reactive epitope to other joint antigens.^55^ The joint environment also promotes local B cell expansion and autoantibody development. Follicular dendritic cell networks in the inflamed synovium exacerbate aberrant B cell and T cell activation.^56^ Impaired regulatory T cell function contributes to loss of self‐tolerance. Many susceptibility genes also modify RA risk and severity.^57^

In summary, molecular mimicry provides a compelling possible mechanism by which microbial agents like EBV could spark generation of the initial self‐reactive lymphocytes involved in RA pathogenesis. Exposure to cross‐reactive foreign antigens sets the stage for inflammatory autoimmunity targeting joint tissues. However, progression to chronic arthritis involves multiple factors including epitope spreading, immune cell infiltration, inflammatory cytokine production, osteoclast activation, and synovial pannus formation. These collectively drive the persistent joint damage and disability characteristic of RA. While more work is needed to prove causality in humans, molecular mimicry represents an important mechanistic model to explain the earliest breach in self‐tolerance leading to joint‐specific autoimmunity. Elucidating these triggers may ultimately inform antigen‐specific immunotherapies for RA.

Ongoing efforts are focused to develop antigen‐specific immunotherapies for rheumatoid arthritis, though it remains an emerging area of research. Researchers are exploring antigen‐specific apheresis to remove rheumatoid factor and ACPA to reduce inflammation.^58^ Vaccines using citrullinated peptide antigens or autologous dendritic cells pulsed with synovial autoantigens are under development to restore tolerance.^59^ Therapies targeting several B cell and T cell epitopes on rheumatoid arthritis‐associated autoantigens are being studied.^60^ Peptide immunotherapy to tolerize T cells reactive against joint‐specific epitopes is also being researched. Some groups are developing synthetic peptides or multi‐antigenic constructs that induce regulatory T cells against arthritis‐associated autoantigens.^61^ Researchers are also exploring ways to selectively delete or anergize autoreactive B cells and T cells.^62^

## 8 SLE

SLE is a complex autoimmune disease characterized by the production of autoantibodies against nuclear antigens and immune complex formation, leading to systemic inflammation and multiorgan damage.^63^ Both environmental and genetic factors contribute to the aberrant adaptive immune responses against self in SLE pathogenesis.^64^ Molecular mimicry between EBV antigens and self‐proteins has been proposed as a mechanism that could initiate the loss of B cell tolerance and autoantibody production.

Evidence indicates EBV infection may be linked to lupus onset and flares. EBNA‐1 contains regions of homology with the Smith (Sm) and Ribonuclear Protein (RNP) autoantigens targeted in SLE.^65^ Through molecular mimicry, immune responses against EBNA‐1 could generate cross‐reactive antibodies and T cells that recognize Sm/RNP and drive autoimmunity.^66^ Anti‐EBNA‐1 antibodies from lupus patients have shown reactivity towards Sm/RNP.^67^ Other EBV proteins also share epitopes with additional SLE autoantigens like Ro 60 kDa.^68^

According to the molecular mimicry model, EBV infection in predisposed individuals leads to polyclonal B cell activation and stimulation of autoreactive B cells expressing surface immunoglobulin receptors specific for self‐nuclear antigens.^69^ Autoreactive helper T cells activated by viral mimicry provide cognate T cell help to these self‐reactive B cells. Immune complexes formed by autoantibodies binding nuclear antigens trigger inflammatory responses and activate dendritic cells, which further promote T cell‐dependent B cell hyperactivity and autoantibody production.^70^

While molecular mimicry may initiate the early stages of SLE pathogenesis, other factors drive perpetuation and clinical progression. Defects in apoptotic cell clearance lead to release of nuclear material that serves to further expand autoreactive lymphocyte clones.^71^ Impaired negative selection and immunoregulation also contribute to continuous activation of self‐reactive B cells and T cells.^72^ Production of IFN‐alpha by plasmacytoid dendritic cells promotes B cell hyperactivity and differentiation into plasma cells secreting high‐titer autoantibodies like anti‐dsDNA, anti‐Sm, and anti‐RNP.^73^

Immune complex deposition in tissues triggers release of inflammatory mediators like complement proteins, IL‐1, IL‐6, and chemokines that recruit leukocytes and propagate localized inflammation and tissue injury.^74^ Common disease manifestations like arthritis, serositis, nephritis, and dermatitis reflect this inflammatory damage in affected organs.^75^, ^76^ While molecular mimicry provides a plausible trigger for initiation of autoreactive responses, propagation of chronic autoimmunity in SLE involves complex interplay between adaptive immunity, innate immunity, and the interferon pathway.

In summary, exposure to EBV viral antigens that mimic self‐nuclear proteins may be an important early step in breaching B cell tolerance in SLE. Molecular mimicry can provide a mechanism for generating the initial cross‐reactive B cells and T cells targeting Smith, RNP, and other lupus autoantigens. However, progression to pathogenic autoantibody production and clinical disease requires additional genetic and environmental co‐factors. These stimulate immune dysregulation, IFN‐alpha production, epitope spreading, plasma cell differentiation, and cytotoxic inflammation induced by immune complexes.^77^ While more proof is still needed, molecular mimicry represents a compelling model to partly explain the genesis of autoreactive lymphocytes central to SLE pathogenesis. Understanding these early triggers may ultimately lead to antigen‐specific therapies.

Vaccines using synthetic peptides or DNA encoding autoantigens like dsDNA, Sm, histones, and ribonucleoproteins are being tested to induce B cell and T cell tolerance.^78^ Approaches using nanoparticles or tolerogenic adjuvants to deliver SLE autoantigens in an immunomodulatory fashion are under investigation.^79^ Some groups are developing B cell epitope peptides that induce a regulatory response against pathogenic anti‐dsDNA and other SLE‐associated autoantibodies.^80^ Based on the molecular mimicry hypothesis, a few preclinical studies are exploring whether vaccination with EBV antigens like EBNA‐1 could desensitize cross‐reactive B cells and T cells.^81^ Dendritic cell vaccines pulsed with nucleosomal antigens are being evaluated for their ability to delete or anergize autoreactive T cell clones.^82^ Drugs that target B cells reactive against disease‐specific epitopes on autoantigen‐antibody immune complexes are under development.^83^ Identifying ways to eliminate or suppress only the subsets of B cells and T cells reactive to lupus‐associated autoantigens remains a major goal.

## 9 GBS

GBS is an acute autoimmune disorder affecting the peripheral nervous system. It is characterized by inflammatory demyelination and axon damage leading to rapidly progressive paralysis and sensory loss. In most cases, GBS follows infection or other immune stimulation that triggers an aberrant autoreactive response targeting nerve components.^84^ Molecular mimicry between microbial antigens and peripheral nerve proteins is a major proposed mechanism for the autoimmunity in GBS.^85^

An association between Campylobacter jejuni enteritis and GBS helped establish the concept of molecular mimicry in GBS pathogenesis. C. jejuni expresses surface lipooligosaccharides that mimic human gangliosides present in peripheral nerve fibers.^86^ Antibodies generated against the bacterial lipooligosaccharides cross‐react with gangliosides like GM1 and GD1a in peripheral nerves, due to shared oligosaccharide epitopes.^87^ This mimicking of nerve gangliosides allows C. jejuni to induce potentially pathogenic anti‐ganglioside antibodies through molecular mimicry.^88^

Other precedential infections, including cytomegalovirus and Mycoplasma pneumoniae, also induce cross‐reactive antibody responses by expressing proteins that mimic nerve glycolipids.^89^ In GBS, these anti‐ganglioside antibodies bind to the nerve axolemma at the nodes of Ranvier and activate complement, provoking inflammatory demyelination.^90^ Macrophage invasion and nerve damage further disrupt saltatory conduction, causing nerve signal dysfunction. In more severe cases, axonal degeneration can occur.^91^

Besides humoral mechanisms, cellular autoimmunity also contributes. Molecular mimicry may prime cross‐reactive T cells that recognize myelin or axonal antigens. These autoreactive T cells and their cytokines contribute to nerve damage and impair remyelination.^91^ Activated T cells infiltrate nerve roots and play a key role in acute motor axonal neuropathy variants of GBS.^92^

In summary, molecular mimicry between microbial antigens and peripheral nerve components provides a mechanism by which infection can trigger autoimmune targeting of nerves in GBS. The immune response intended against the pathogen leads to production of antibodies and T cells that cross‐react with gangliosides and other peripheral nerve proteins. However, clinical manifestation and disease severity depend on other factors like prior exposure history, genetics, and nature of the preceding infection.^93^ While molecular mimicry explains the initial breach in self‐tolerance, the subsequent neuromuscular paralysis reflects immune‐mediated nerve injury involving both cellular and humoral effectors. Demyelination, conduction block, and sometimes axonal degeneration produce the hallmark ascending paralysis.^94^ Further research on the specific molecular mimics involved will help inform antigen‐specific therapies for treating GBS in the future.

Recent studies are evaluating removal of anti‐ganglioside antibodies using immunoadsorption columns or dialysis as a treatment approach^95^ since plasmapheresis or plasma exchange helps remove some pathogenic autoantibodies.^96^ Some research groups are developing small synthetic antigens or peptides that mimic the ganglioside structures and could selectively bind and neutralize anti‐ganglioside antibodies.^96^ Researchers are also working on assays to detect the presence of antibodies against various ganglioside mimics to help predict GBS risk after infections.^97^ Vaccines using ganglioside‐mimicking antigens are being explored to induce tolerance to these peripheral nerve targets and reduce risk of subsequent autoimmunity.^98^ Studies aim to better characterize cross‐reactive T cells and their myelin or axonal protein targets, which could enable antigen‐specific immunomodulation.^99^ Overall, substantial challenges remain in elucidating the specific molecular targets and pathways involved in GBS. But research continues on leveraging antigen‐specific approaches to predict, prevent, and treat GBS.

## 10 AUTOIMMUNE MYOCARDITIS

Autoimmune myocarditis involves inflammation and damage to heart muscle due to loss of tolerance and autoreactive immune responses targeting cardiac proteins. While multiple factors can trigger myocarditis, molecular mimicry between microbial antigens and cardiac myosin is a proposed mechanism for the initial autoimmune activation.

Several lines of evidence indicate coxsackievirus B may induce myocarditis through molecular mimicry with cardiac myosin heavy chain‐alpha.^100^ Shared peptide sequences result in structural similarity between coxsackievirus proteins and myosin epitopes. This can lead to activation of cross‐reactive T cells by viral antigens that then attack heart muscle expressing myosin.^100^ Studies utilizing mouse models have shown that infection with coxsackievirus can lead to activation of cross‐reactive T cells and subsequent development of myocarditis; these autoimmune effects were able to be prevented by approaches that inhibited the immune responses against cardiac myosin.^101^ Molecular mimicry with other heart proteins like adenine nucleotide translocator, cardiac troponin I, and cardiac mitochondria have also been reported and may expand autoreactive responses through epitope spreading.^102^

According to the molecular mimicry model, mimicry between myocarditic viruses and heart proteins primes self‐reactive T cells. The activated autoreactive CD4+ T helper cells stimulate B cells to produce autoantibodies, and cytotoxic CD8+ T cells directly damage cardiomyocytes expressing the mimicked antigens.^103^ Local release of cytokines like IFN‐gamma, TNF‐alpha, and IL‐1beta amplifies immune‐mediated myocyte necrosis and cardiac inflammation.^103^ Activated macrophages and neutrophils infiltrate the myocardium, secreting more inflammatory mediators.^104^

While molecular mimicry triggers the initial autoimmune reaction, other factors perpetuate disease. Epitope spreading expands T cell reactivity from the original viral mimic to additional cardiac antigens beyond myosin. Bystander activation of T cells reactive to unmodified cardiac proteins also occurs.^105^ Ongoing cardiac cell damage provides autoantigenic stimuli that further drive expansion and differentiation of autoreactive B and T cell clones.^106^ Imbalance between regulatory and effector T cell activity impairs peripheral tolerance.^107^

In summary, homologies between coxsackievirus or other cardiotropic viruses and myosin may lead to activation of cross‐reactive T cells through molecular mimicry that initiate autoimmune pathology in the heart. However, progression from initial autoreactive response to clinically evident myocarditis requires loss of normal tolerance mechanisms like regulatory T cells. Understanding these complex interactions between environmental triggers and genetic susceptibility is key to deciphering pathogenesis. Elucidating the specific mimicry‐inducing antigens could ultimately guide development of targeted immunotherapies.

Some preclinical studies are exploring coxsackievirus vaccines using viral capsid proteins or inactivated virus to protect against myocarditic strains and reduce molecular mimicry triggers.^108^ Researchers are also investigating whether immunization with cardiac myosin peptides can induce tolerance in animal models and prevent autoimmune myocarditis.^109^ Identifying immunodominant T cell epitopes on myocarditic viruses and their homologous cardiac antigens could enable antigen‐specific immunotherapy to selectively tolerize those cross‐reactive T cells.^110^ Determining susceptibilities like HLA associations may help personalize antigen‐specific prevention or treatment based on an individual's molecular mimicry risk.^111^

## 11 PBC

PBC is an autoimmune liver disease characterized by chronic inflammatory destruction of the small intrahepatic bile ducts, leading to cholestasis, fibrosis, and cirrhosis. Both cellular and humoral immunity contribute to bile duct injury and loss. Autoantibodies and T cells target mitochondrial and nuclear antigens expressed in biliary epithelial cells. Molecular mimicry between microbial proteins and self‐antigens has been proposed as a mechanism that could spark autoreactive responses.

Several lines of evidence indicate molecular mimicry may be involved in PBC pathogenesis. Serum antibodies from PBC patients cross‐react with both the E2 component of pyruvate dehydrogenase complex (PDC‐E2) and bacterial proteins with similar sequences. The dominant autoepitope on PDC‐E2 shares homology with common gut bacteria, possibly inducing cross‐reactivity through mimicry.^112^ Cholangiocytes also express Toll‐like receptors that could be activated by microbial mimic antigens.^113^

According to the mimicry hypothesis, exposure to microbes with antigens that mimic mitochondrial or nuclear components of biliary cells provokes activation of cross‐reactive B cells and T cells targeting those self‐proteins. Autoreactive CD4+ and CD8+ T cells infiltrate the liver and orchestrate an inflammatory response against small bile ducts expressing the mimicked antigens.^114^ Activated macrophages, natural killer cells, and neutrophils potentiate bile duct damage.^115^ Autoantibodies like anti‐mitochondrial antibodies (AMA) further promote bile duct apoptosis.^116^

However, perpetuation of chronic biliary autoimmunity likely involves more than just molecular mimicry as the initial trigger. Local inflammatory mediators, deficiency in immune regulation, and biliary epithelial stress from cholestasis may amplify and spread the autoreactivity.^117^ Genetic risk factors such as certain HLA alleles shape susceptibility. Potentially, an environmental trigger like a mimic antigen instigates immune activation, but other genetic and nongenetic cofactors interact to facilitate loss of tolerance to biliary proteins and progression to PBC.^118^

In summary, molecular mimicry between microbial proteins and self‐antigens like PDC‐E2 provides a plausible mechanism by which infection or dysbiosis could precipitate the early stages of PBC pathogenesis. Exposure to a bacterial, viral, or fungal mimic could initiate activation of biliary‐reactive lymphocytes. However, the chronicity of PBC suggests additional factors perpetuate the autoimmune biliary damage over time. Ongoing research to better characterize the inciting mimicry antigens and corresponding autoantigen targets may enable antigen‐specific immunomodulatory therapies in the future. Elucidating other risk factors and disease pathways is also key to devising strategies to restore tolerance.

## 12 POTENTIAL CLINICAL IMPLICATIONS OF MOLECULAR MIMICRY‐INDUCED AUTOIMMUNITY

The concept of molecular mimicry has significant clinical implications if proven to be a major mechanism underlying autoimmunity. The cross‐reactivity arising from mimicry between exogenous and self‐antigens is hypothesized to be one pathway leading to loss of tolerance and development of autoimmunity. Elucidating the role of molecular mimicry in human autoimmune conditions could impact prevention, diagnosis, and treatment.

One significant clinical implication is the potential to identify the specific mimicry‐inducing antigens responsible for precipitating autoimmunity in susceptible individuals. If the microbes and microbial proteins that trigger cross‐reactive autoimmune responses can be pinpointed, it may enable the prediction of an individual's risk based on their exposure history. For example, infection with a particular strain of streptococcus or influenza virus could be associated with an increased future risk of autoimmunity based on the molecular characteristics of that pathogen. Identifying the precipitating mimic antigens may also allow for more personalized diagnosis, rather than just categorizing patients based on disease phenotype. This could significantly improve prognostic capabilities.

Another implication is the development of molecular mimicry‐based biomarkers to monitor autoimmune progression or flares. Once the critical self‐antigens involved in a mimicry‐mediated autoimmune response are known, antibodies against those self‐antigens could be tracked as disease biomarkers in at‐risk individuals. Rising titers may indicate more significant autoimmune activity before substantial clinical symptoms manifest. Biomarker screening could enable earlier intervention to minimize tissue damage from emerging autoimmunity.

Based on molecular mimicry insights, strategies targeting the inciting microbial pathogen, or its disease‐driving antigens can also be envisioned targeting the inciting microbial pathogen. For instance, vaccines against the microbes displaying specific mimicry epitopes may reduce autoimmune risks by preventing the infections that initiate cross‐reactivity. Therapies that selectively inhibit the activation of immune cells reacting against those epitopes could also be feasible. Such antigen‐specific immunotherapies may effectively dampen autoimmune responses while avoiding general immunosuppression.

Additionally, harnessing knowledge about molecular mimicry pathways may facilitate the development of therapeutic self‐antigen vaccines or peptides. Carefully administered self‐antigen derivatives may promote the rebuilding of tolerance against over‐reactive immune cells. Current research already explores this approach in MS, where myelin‐mimicking epitopes are used to tolerate autoreactive T and B cells. Similar strategies informed by molecular mimicry mechanisms could yield clinical benefits for other autoimmune disorders. Furthermore, elucidating the genetic factors predisposing specific individuals to mount sustained autoimmune reactions against mimic antigens could enable better risk profiling. Genetic screening may 1 day identify those most vulnerable to disease after specific environmental exposures. This could lead to personalized preventive measures. A detailed understanding of gene‐environment interactions is critical to translating molecular mimicry concepts into clinically predictive tests.

A summary of some proposed examples of molecular mimicry between microbial pathogens and self‐antigens involved in autoimmune diseases is shown in (Table 1). The table includes the name of the human host protein, corresponding antigen name, pathogen exhibiting molecular mimicry, specific pathogen protein containing the mimic epitope, taxonomic classification of the pathogen, sequence of the pathogenic epitope, associated autoimmune condition, and literature reference.

**Table 1:** Amino acid sequence of pathogenic epitopes and their human counterparts in different autoimmune disease models.

Autoimmune diseases	Host protein	Host antigen	Pathogen	Pathogenic protein	Pathogen taxonomy	Pathogenic epitope	Ref
Myocarditis	Cardiac myosin	LEDLKRQLEEEVKAKNA	Group A streptococcus	Streptococcal M5 protein	Gram positive bacteria	TIGTLKKILDETVKDKIA	[119]
Myocarditis	Cardiac myosin	KLQTENGE	Group A streptococcus	Streptococcal peptide NT4	Gram positive bacteria	GLKTENEGLKTENEGLKTE	[119]
Type‐1 DM	GAD65	SIMAARYKYFPEVKTKGMAAVPKL	Coxsackievirus	P2_CproteinofCoxsackievirusB4	ssRNA viruses	FIEWLIKVKILPEVKEKHEFLSRL	[120]
Type‐1 DM	GAD67	AMMIARFKMFPEVKEKGMAALPRL	Coxsackievirus	P2_CproteinofCoxsackievirusB4	ssRNA viruses	FIEWLIKVKILPEVKEKHEFLSRL	[120]
Type‐1 DM	GAD65	IAFTSEHSHFSLK	Rubella virus	RVE1	ssRNA viruses	RVKFHTETRTVWQLSVAGVSC	[121]
SLE	Ro	TKYKQRNGWSHK	Epstein Barr virus	Epstein_Barr virus nuclear antigen_1 (EBNA_1)	dsDNA viruses	GGSGSGPRHRDGVRR	[122]
SLE	SmD	PPPGMRPP	Epstein Barr virus	Epstein_Ban nuclear antigen_1 (EBNA_1)	dsDNA viruses	PPPGRRP	[122]
MS	MBP	VVHFFKNIVTP	Schizosaccharomyces pombe	Protein kinase CHK1	Fungi	WRKFFKNVVSS	[122]
MS	MBP	VVHFFKNIVTP	Human cytomegalovirus	Tegument Protein UL71	dsDNA viruses	DILILKLVVGE	[122]
MS	MBP	VVHFFKNIVTP	Salmonella typhimurium	UDP_N_acetylenolpyruvoyl_glucosamine reductase	Gram negative bacteria	AGSFFKNPVVA	[122]
MS	MBP	ENPVVHFFKNIVTPR	*Staphylococcus aureus*	VgaB	Gram positive bacteria	VLARLHFYRNDVHKE	[123]
MS	MBP	ENPVVHFFKNIVTPR	Mycobacterium avium	Transposase	Gram positive bacteria	QRCRVHFLRNVLAQV	[123]
MS	MBP	ENPVVHFFKNIVTPR	Bacillus subtilis	YqeE	Gram positive bacteria	ALAVLHFYPDKGAKN	[123]
MS	MBP	ENPVVHFFKNIVTPR	Herpes simplex virus	UL15 protein	dsDNA viruses	FRQLVHFVRDFAQLL	[124]
RA	HSP60	HRKPLVIIAEDVDGE	Mycobacterium bovis	HSP65	Gram positive bacteria	AGKPLLIIAEDVEGE	[125]
PBC	PDC_E2	KLSEGDLLAEIETDK	Lactobacillus delbrueckii	BGAL	Gram positive bacteria	RDSEGDLVAEKLGPI	[126]

A graphical protein sequence alignment was generated using “NCBI Blast: https://www.ebi.ac.uk/Tools/services/rest/ncbiblast” to compare the amino acid sequence “QRCRVHFLRNVLAQV” from Mycobacterium avium to human GCP4 (Gamma‐tubulin complex component 4), a major myelin protein from the CNS. The alignment shows a region of sequence homology spanning residues 85−95 in the Mycobacterium protein and residues 103−113 of the GCP4 sequence. This homologous peptide sequence is highlighted in the graphical alignment, indicating a sequence mimic between Mycobacterium avium and the human myelin protein. Molecular mimicry based on this sequence similarity may potentially contribute to autoimmune responses targeting myelin in MS.

While promising, substantial work remains to validate molecular mimicry as a pathogenic process in human autoimmune conditions and harness it for therapeutic innovations. Overcoming current limitations in establishing definitive causative relationships is critical. However, the clinical payoffs make this a worthwhile research direction. Even moderate progress in leveraging molecular mimicry for improved prediction, monitoring, and treatment of autoimmune disorders would be impactful. In summary, molecular mimicry as a presumptive trigger for loss of self‐tolerance has far‐reaching clinical implications that warrant rigorous investigation to transform this long‐standing theory into medical applications.

## CONFLICT OF INTEREST STATEMENT

The author declare no conflict of interest.

## Data Availability

Data sharing not applicable to this article as no datasets were generated or analyzed during the current study.
